# Contemporary Role and Applications of Artificial Intelligence in Dentistry

**DOI:** 10.12688/f1000research.140204.1

**Published:** 2023-09-20

**Authors:** Talal Bonny, Wafaa Al Nassan, Khaled Obaideen, Maryam Nooman Al Mallahi, Yara Mohammad, Hatem M. El-damanhoury

**Affiliations:** 1 Department of Computer Engineering, University of Sharjah, Sharjah, 27272, United Arab Emirates; 2 Sustainable Energy and Power Systems Research Centre, RISE, University of Sharjah, Sharjah, 27272, United Arab Emirates; 3 Department of Mechanical and Aerospace Engineering, United Arab Emirates University, Al Ain City, Abu Dhabi, 27272, United Arab Emirates; 4 ‎College of Engineering and Information Technology, Ajman University, Ajman University, Ajman, Ajman, United Arab Emirates; 5 ‎Department of Preventive and Restorative Dentistry, College of Dental Medicine, University of Sharjah, Sharjah, 27272, United Arab Emirates

**Keywords:** Artificial Intelligence, Digital Dentistry, Dentistry, Deep Learning, Artificial Neural Networks, ‎Dental Specialty

## Abstract

Artificial Intelligence (AI) technologies play a significant role and significantly impact various sectors, including healthcare, engineering, sciences, and smart cities. AI has the potential to improve the quality of patient care and treatment outcomes while minimizing the risk of human error. Artificial Intelligence (AI) is transforming the dental industry, just like it is revolutionizing other sectors. It is used in dentistry to diagnose dental diseases and provide treatment recommendations. Dental professionals are increasingly relying on AI technology to assist in diagnosis, clinical decision-making, treatment planning, and prognosis prediction across ten dental specialties. One of the most significant advantages of AI in dentistry is its ability to analyze vast amounts of data quickly and accurately, providing dental professionals with valuable insights to enhance their decision-making processes. The purpose of this paper is to identify the advancement of artificial intelligence algorithms that have been frequently used in dentistry and assess how well they perform in terms of diagnosis, clinical decision-making, treatment, and prognosis prediction in ten dental specialties; dental public health, endodontics, oral and maxillofacial surgery, oral medicine and pathology, oral & maxillofacial radiology, orthodontics and dentofacial orthopedics, pediatric dentistry, periodontics, prosthodontics, and digital dentistry in general. We will also show the pros and cons of using AI in all dental specialties in different ways. Finally, we will present the limitations of using AI in dentistry, which made it incapable of replacing dental personnel, and dentists, who should consider AI a complimentary benefit and not a threat.

## Introduction

Artificial intelligence (AI) is the process of training a computer in such a way that it starts thinking like human beings. ^1^ With the advancement in technology in AI, the idea behind the machine that performs tasks for human beings is becoming old-fashioned. Devices and systems that can perform basic calculations and fundamental tasks or detect the text with various recognition applications are now considered standard computer applications and does not lie under AI. This globally recognized technique constantly improves many sectors, including medical, engineering, ^2^^–^^6^ energy, ^7^^,^^8^ mechanical, ^9^ computer science, ^10^^,^^11^ psychology, ^12^ and other disciplines. ^13^^–^^19^ This constant improvement in AI makes it an important constituent of almost every field. Along the same line, AI is one of the enablers of achieving the United Nations Sustainable Development Goals (SDGs). ^20^ More specifically, AI could accelerate the achievement of SDG 3: Good Health and Well-being. ^21^^,^^22^

In dentistry, AI is becoming an important constituent owing to its use in improving the diagnostic facility to provide excellent patient care and the ability to get excellent results. ^23^^–^^26^ Dentists are capable enough with their training to analyze and suggest the best possible treatment and provide the best clinical decision based on their experiences. ^27^^,^^28^ However, sometimes, dentists do not have sufficient information and knowledge to predict the best clinical practice. ^29^ This may result in poor clinical decision-making and bad effects on human well-being. ^30^ However, the dentist can perform better using AI systems by making accurate decisions. ^31^ They can rely on computer and AI software to make clinical decisions. ^32^^,^^33^ Several papers intend to explore and review the role of AI in dentistry. ^34^^,^^35^ However, most of these papers were very technical or focused on the technical perspective of AI's application in dentistry, making them quite difficult for dental practitioners.

This paper attempts to review the solutions used in digital dentistry that engineers have developed from various fields of knowledge. As a result, this knowledge is critical for dentists, surgeons, clinicians, and laboratory technicians. Moreover, most of the previous papers did not explore the role of AI in different dental specialties; diagnosis, clinical decision-making, treatment, and prognosis prediction in ten dental specialties; dental public health, endodontics, oral and maxillofacial surgery, oral medicine and pathology, oral & maxillofacial radiology, orthodontics and dentofacial orthopedics, pediatric dentistry, periodontics, prosthodontics, and digital dentistry in general, so this paper intends to fill this gap by reviewing the application of AI in dentistry using simplified terms and showing the role of AI in different dentistry specializations. As a result, this paper focuses on answering the following research questions:

• What are the trends, applications, and innovations of AI in dentistry and digital dentistry that are expected to influence the direction of dental research in the coming years?
• What are the strengths and weaknesses of AI in dentistry?
• What are the challenges of using AI in dentistry?

The paper is structured as follows. The next section reviews artificial intelligence and its application in healthcare. The application of AI in dentistry and digital dentistry is then described. The strengths and weaknesses of AI in dentistry are presented in the section after. Then we present the main challenges of using AI in dentistry. Finally, the conclusion is given in the final section.

## Artificial Intelligence (AI) for Healthcare

**Figure 1.**
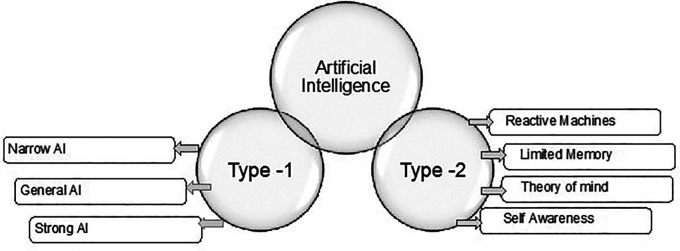
Artificial Intelligence types.

The science of AI contains different branches, such as machine learning (ML) and deep learning (DL). ML is a system that can be trained by different problem-solving training and models to gain the ability to automate the process of solving tasks. ^36^ DL is a part of ML in which the learning module is based on artificial neural networks. ^37^ DL provides an excellent ability to beat state-of-the-art techniques in its application to perform various tasks and analyze and evaluate data from various sources, including audio, ^38^^,^^39^ sensors, ^40^ and visual data. ^41^ On the other hand, there are several types of artificial intelligence. Under two main categories based on their functionalities and capabilities, as shown in Figure 1.

**Figure 2.**
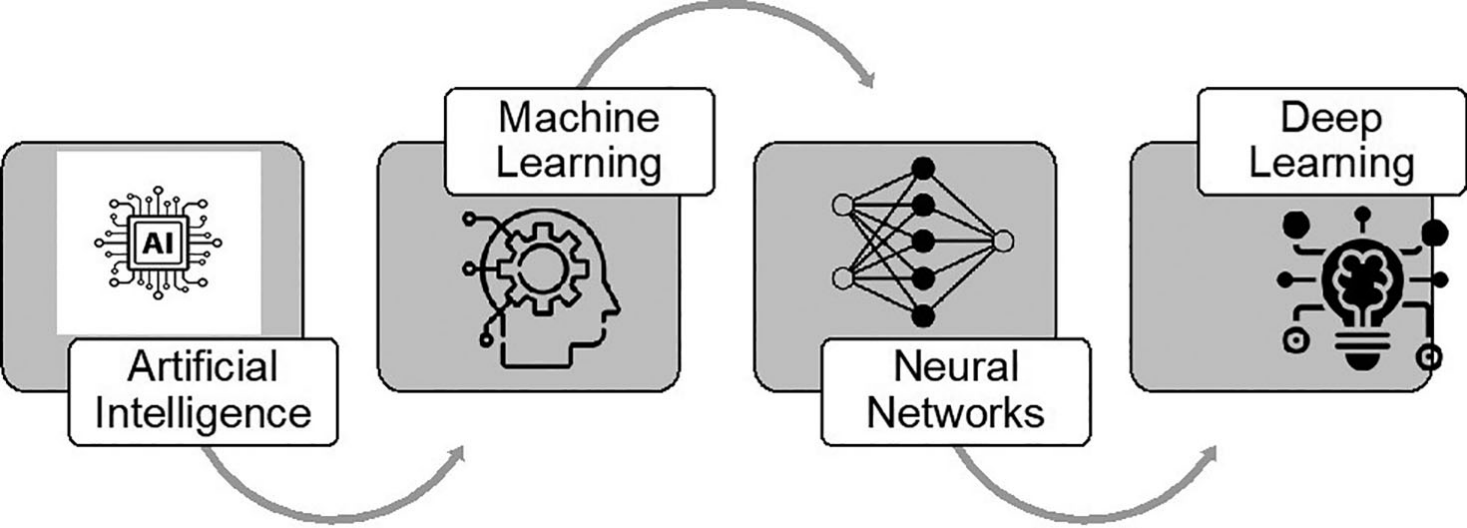
Key aspects of artificial intelligence.

Since its inception, AI has significantly improved our daily lives and activities in various ways. Emerging fields, including surgery, ^42^ automatic disease diagnosis, ^43^ and the recently established personalized medicine, can benefit from AI support. ^44^ Figure 2 demonstrates the key aspects of artificial intelligence, ranging from ML, Neural Networks (NN), and DL.

Moreover, AI models were implemented to central processing unit (CPU) and graphics processing unit (GPU) architectures, so detection\recognition-based algorithms can achieve practical needs. However, recent AI algorithms, especially deep learning models, demonstrated efficiency in solving many problems. Those algorithms demand an intensive CPU and memory, which prevents standard CPUs from achieving the desired performance levels. ^45^ As a result, hardware processors, specifically field-programmable gate arrays (FPGAs) platforms, have been used to effectively maximize those models' performance. ^46^^–^^48^ FPGA is an integrated circuit customized for a specific application where the configuring process is applied after manufacturing. ^49^ Consequently, FPGA is the perfect candidate for implementation from various perspectives, including but not limited to performance for parallel tasks, cost-effectiveness, prototyping, and real-time applications. ^50^^,^^51^

## Application of AI in Dentistry

Artificial intelligence has a large number of advantages in various businesses as well as various domains. In healthcare, AI has been getting attention in the medical and pharmaceutical fields by providing self-driven operating applications. AI applications in the healthcare domain include Robotic systems, Diagnostic Aids, and Drug Discovery. ^52^^,^^53^

AI emerged in the healthcare field to "train" computer programs to reach highly intelligent capabilities. AI, a superior diagnostic aid that minimizes daily practice mistakes, might enhance patient care. ^54^ The interpretation of medical images has progressed from expert systems through atlas-based models toward the potential of deep learning. The huge data from digital radiographs may be utilized since it has much potential for improving radiology diagnosis with AI. Through automated data mining, AI with DL might help medical radiology. ^55^ New information will be found with only a little human understanding. ^56^^–^^59^

The identification and management of systemic conditions during dental treatment has always been a challenge to the dentists. AI can examine electronic medical records and scientific datasets practically and efficiently. With the advent of huge scientific data, the diagnosis of human congenital abnormalities has improved. In medical research, the support vector machine (SVM), a machine-learning model, has become a typical way of analysis. SVM may be used to classify complex situations like traumatic brain damage. ^60^^,^^61^ DL may play a crucial role in advancing cardiovascular medicine due to the requirement for precision in diagnosis and therapy. ^62^ With the adoption of deep learning, it will be feasible to identify numerous cardiovascular disorders with high-precision diagnosis. ^63^ Wearable technology combined with intelligence applications can anticipate a patient's life-threatening crisis such as a stroke, allowing physicians to deliver effective, early therapies. ^54^ Using AI to examine viruses has boosted transitional studies in viral immune monitoring. ^64^

AI can help fill knowledge gaps while also lowering costs and increasing benefits. AI is commonly utilized in disease management to examine treatment outcomes and provide precision medicine. These cutting-edge machine-learning algorithms are powerful analytic tools that assist clinicians in understanding and analyzing moods.

**Figure 3.**
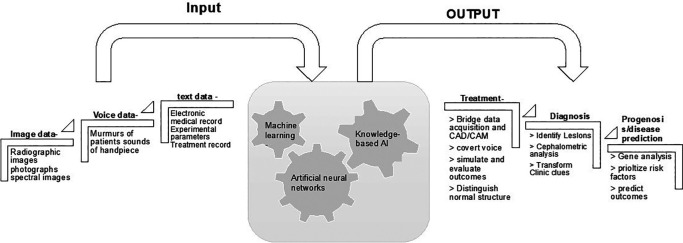
Summary of the hierarchy and major dental applications of AI.

Traditional components of dentistry have been modernized by using AI. In dentistry, AI-based diagnostic systems and patient data are widely used. They are mostly decision systems based on clinical knowledge, which leads to helping and advising specialists in making better judgments. This highly efficient technology has been employed to analyze treatment planning, diagnosis, and prognosis prediction. Because of their usefulness in offering explanations and logic, these systems are in high demand. AI has transformed dentistry, resulting in reducing the work of a dentist. ^65^ AI-based technology in dentistry is mainly aimed at providing expert assistance to healthcare providers. ^66^^–^^68^ Any computer software created to assist health professionals in making clinical choices and dealing with medical knowledge essential for understanding such data is referred to as a clinical decision support system. ^32^ Figure 3 displays the structure and major dental applications of AI.

**Figure 4.**
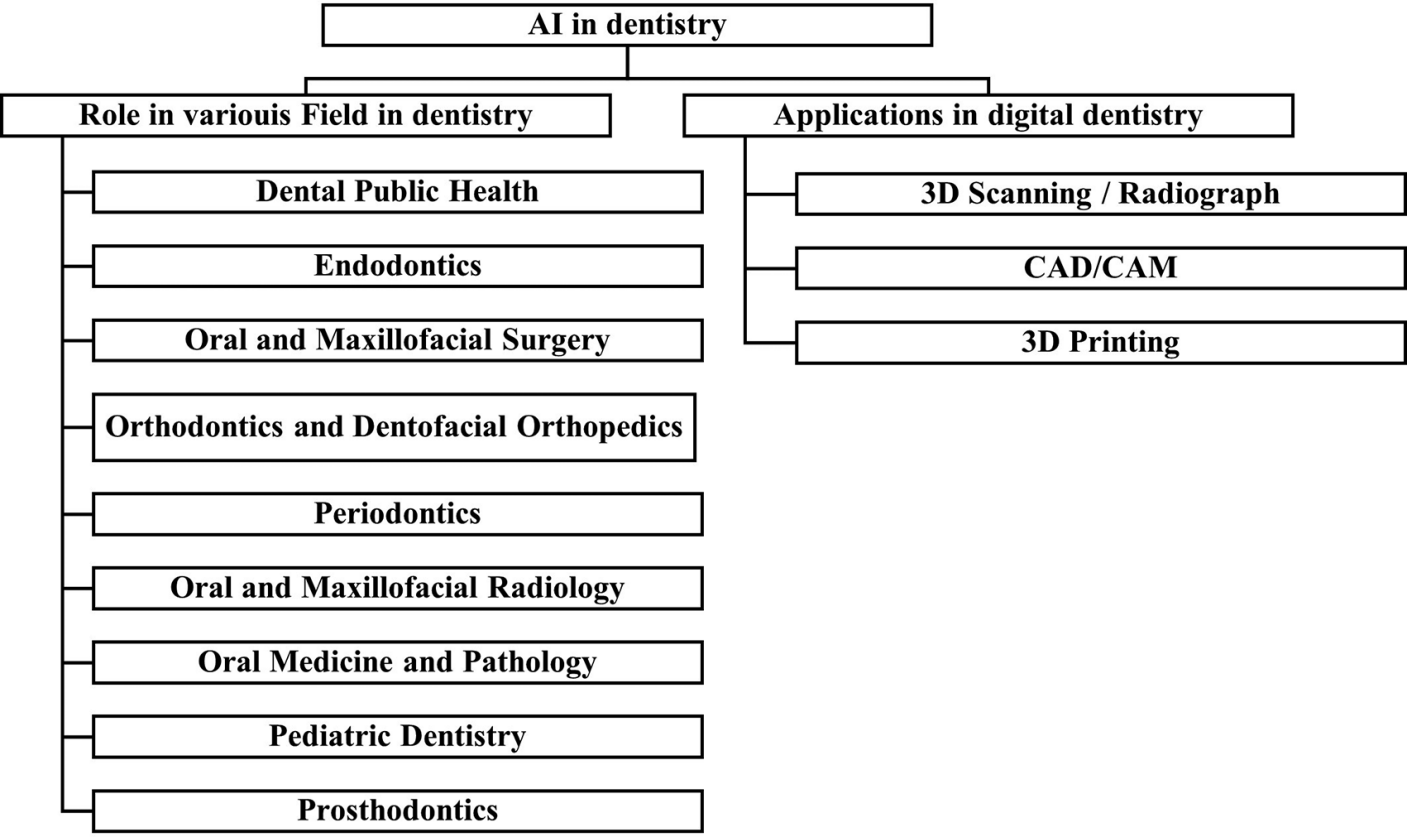
The categories of AI applications in dentistry.

After reviewing various scientific journals, key information such as applications, AI techniques, and field of research was investigated and categorized, as shown in Figure 4.

### State-of-the-art AI technology in Dentistry

This section describes the state of the art of AI technology in the dentistry field. AI provides essential help to automate different activities, such as the assessment of a radiographic image to detect a dental disease faster and more accurately than other activities. It has been shown how appropriately trained algorithms can be extremely successful for the diagnostician and how AI can determine the direction of public health and clinical services.

### ANNs

Artificial neural networks (ANN’s) are systems based on algorithms, learning on a neural network with biological functionality. Such systems address various challenges, including supervised, unsupervised, and reinforcement learning. An ANN's fundamental structure is a series of data layers interconnected by operational neurons. The most promising area of study in this discipline is undoubtedly DL applications in dentistry. Because these approaches allow discovering particular arrangements from massive data sets of pictures, they can help build a high-performance system with excellent decision-making capabilities. ^69^

Although it may not appear that advances in AI have had a significant impact on dentistry, various areas, including automated detection of oral diseases, enhancement and analysis of dental x-rays and images, are some of the benefits of AI in dentistry. On the robotics front, various advancements are allowing robotic assistance in dentistry. ^70^

Several variables have played a role in the latest AI revolution in biomedicine. For starters, data collection has grown exponentially over the past few decades. However, data alone is insufficient. New sophisticated AI algorithms have permitted a complete and helpful extraction of information from acquired data because of improvements in high-performance computing (HPC). This information extraction technique is called ML, the data-driven component of AI that seeks to enable machines (algorithms implemented in computer systems) to learn about a specific topic from a specific dataset. ^71^ This sort of information extraction is often carried out utilizing supervised learning approaches, which have proven to be quite effective in solving many issues. Supervised learning aims to train a function that converts an input sample to the desired output using a dataset of input-output pairings. After learning this function using the training dataset, new predictions can be formed over incoming samples. ^72^

These technologies have also seen substantial medical adoption, namely in computer vision. A variety of factors has been recognized as driving this adoption. Diagnostic imaging is critical in many healthcare disciplines, ^73^ and AI is particularly well suited to overcoming the diversity in individual subjective inspection and increasing the effectiveness of therapy while cutting costs by removing mundane activities. Digital health data are being collected everywhere, and while these data are currently somewhat heterogeneous, businesses are progressively aiming to deliver cleansed, curated, and organized data. ^74^

The convolutional neural network (CNN) is the ANN's most often utilized subclasses in medicine and dentistry. ^75^ A CNN employs a unique neuron connection architecture and the mathematical function convolution to handle digital data such as sound, pictures, and videos. CNNs evaluate a larger picture or signal by scanning a small neighborhood of inputs simultaneously, from left to right and top to bottom, using a sliding window. They are the most often used image recognition method because they are particularly well matched to picture categorization. ^76^

### Augmented Reality and Virtual Reality

Dental care is a one-of-a-kind career in the medical field, and it is incredibly required as it is a necessity hence the incorporation of a vast quantity of data and the development of clinical skills. ^77^ A technology that superimposes a computer-generated picture over a user's perspective of the actual world, producing a composite vision, according to the definition of augmented reality. ^78^ The development of augmented reality has generated appealing prosthetics resulting in better and improved overall patient experiences. The patient can try on the augmented reality that can be altered until they are satisfied using AI algorithms and augmented reality. The final prosthesis is produced precisely to these specifications. ^79^^,^^80^

Irrespective of augmented reality, virtual reality is a simulation based on a 3D picture generated by the computer, which can interact with the real and physical world and electrical and electronic equipment. In combination with virtual reality, AI systems have been utilized to alleviate dental concerns and as a strong tool for treating a patient with pain that cannot be cured by medicine. ^81^

The field of intelligent teaching organizations has advanced greatly since its inception in the 1980s. To create scenarios that simulate clinical work on patients while removing the risks associated with teaching a live patient, augmented reality and virtual reality are both extensively used in dentistry education. ^82^ The preclinical virtual patient has greatly improved the quality of feedback provided since AI was recently included in intelligent teaching systems, namely the Unified Medical Language System (UMLS). In the interactive interphase, students can evaluate their work and compare it to the ideal, resulting in high-quality training settings. Studies has highlighted the importance of these systems and reported that students achieve competency-based skill levels faster than those using standard simulator modules. ^83^^,^^84^

### Data Mining

While ML makes predictions based on an analytical approach to available data, data mining is concerned with discovering causal links and similarities in current data. Data mining of digitized dental data allows for the analysis of variance across dentists when detecting and predicting dental caries, dental age estimation. ^33^^,^^85^^–^^87^

### Applications in the Various Fields of Dentistry

**Figure 5.**
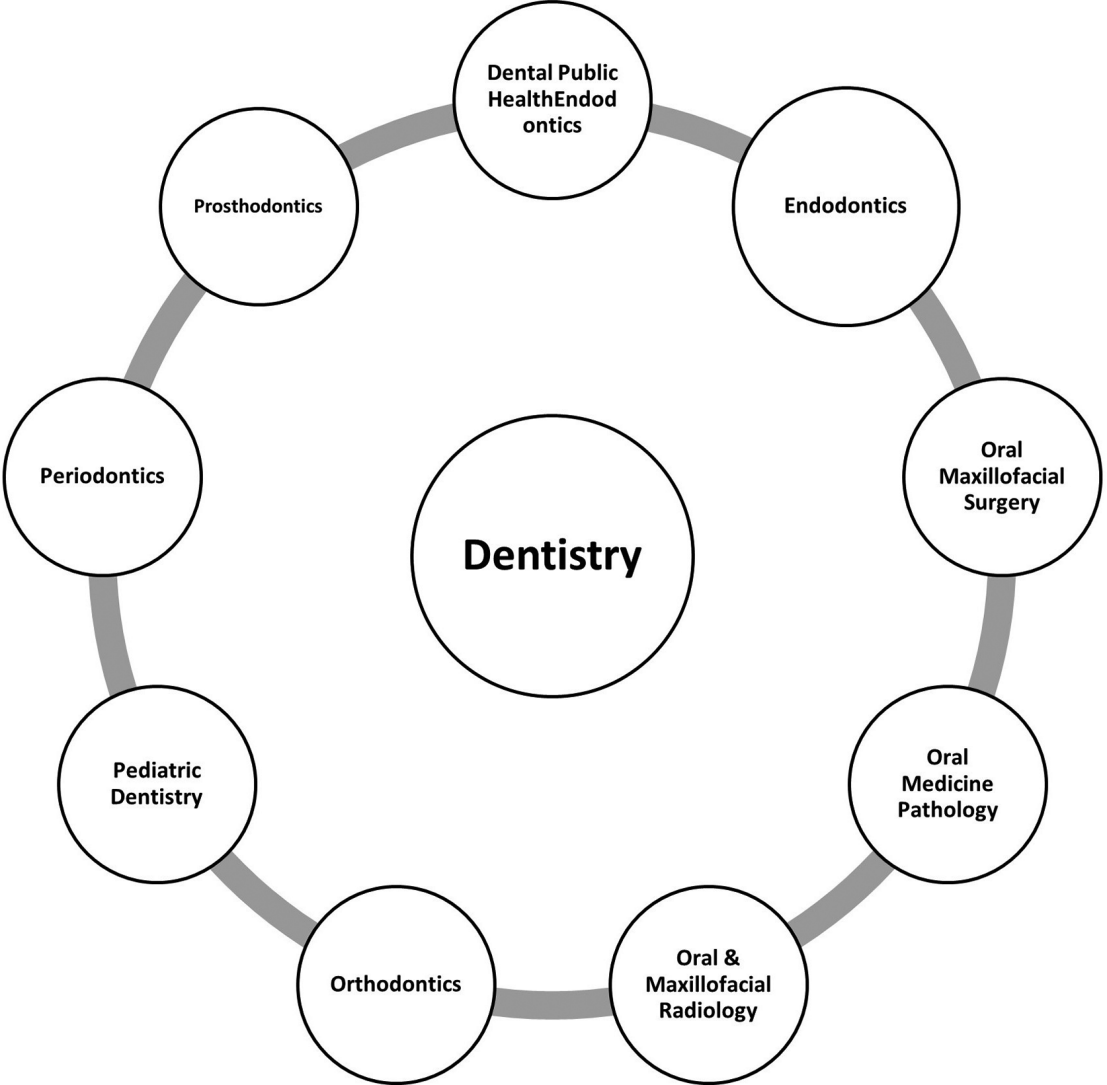
Dental Specialties based on Canadian Dental Association.

Dental public health, endodontics, oral and maxillofacial surgery, oral medicine and pathology, oral and maxillofacial radiology, orthodontics and dentofacial orthopedics, pediatric dentistry, periodontics, and prosthodontics are dental specialties recognized by the Canadian Dental Association as shown in Figure 5. ^88^

Virtual dental assistants working on AI are now working for various domains in the market. Like virtual assistants, these designed systems can perform specific tasks in dental clinics more precisely and accurately, using less power, resulting in less error than human beings. ^80^

Some of the virtual assistant work in the field of dentistry includes,

▪ Offering the patient a convenient appointment time reduces the potential for commotion and activity arising due to the large number of patients coming in a day. ^89^
▪ Informing the dentist and the patients regarding the upcoming checks when hereditary or lifestyle factors suggest a higher vulnerability to oral problems. (e.g., diabetic patient's periodontal screening and users of smoked and smokeless tobacco regularly will be tested for oral cancer screening). ^90^
▪ Taking care of paperwork and insurance.
▪ Helping in better diagnosis and planning after the patient's disease diagnosis is carried out.
▪ Provide the dentist with information about the allergies and diseases the patient already has before the patient visit.
▪ Providing the dentist with the associate medical background. This will help the doctor finalize the plan regarding the patient's diagnosis. This also helps in planning surgery for the patient. ^33^
▪ Offering telephone-based services on time in the cases when the dentist cannot reach the hospital and urgent medical care is required. ^91^

**Figure 6.**
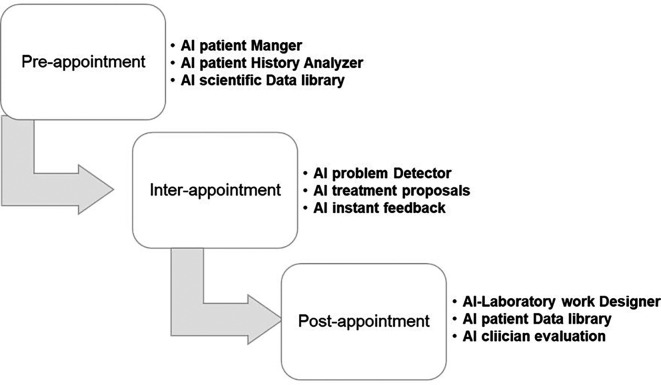
The comprehensive dental care system of the future with AI. It can be divided into pre-appointment, inter-appointment, and post-appointment AI systems. These three types of AI gather data that will be used to enhance patient care.

Figure 6 demonstrates a comprehensive dental care system for the future with AI. Aside from this, AI-based software allows us to develop a comprehensive data history for each patient, which may be incredibly thorough and accessible. ^27^^,^^92^ The AI software can recognize the voice, which will help the dentist improve their performance. AI software can perform documentation and display all the data relevant to the dentist at a rather efficient and faster speed than a human equivalent. (For example, gathering all relevant patient records on their dental visits, supplementary oral pictures, x-rays, and diagnostic graphs related to their disease). Owing to these exceptional learning abilities, it can be trained to execute various tasks. Such devices can be used alongside dental x-rays and CT-scans to detect changes in human teeth from their normal teeth that the dentist's eyes may not have noticed. ^27^^,^^93^

This technique may also be used to reach orthodontic diagnosis by analyzing the cephalometric radiographs and detecting dental and skeletal abnormalities. ^94^ Simply by successfully obtaining the patient's data and synchronizing it with the decision system already in place for the patient in the dental labs, this is accomplished. ^95^ In pathology, scanning numerous sections to find minute details that aid in diagnosis and clinical decision-making is possible. The program can do different analyses of images and radiographs in orthodontics to aid in diagnosis and treatment planning. ^96^ Taking a dental impression might soon be obsolete due to advancements in intra-oral scanners and cameras. These digital impressions minimize all laboratory operations, which sharply lowers the frequency of errors and is quicker and more accurate.

Using AI, the computer can notify the dentist throughout the process of creating a digital imprint and assist in making an excellent impression. ^97^ Taking a dental impression may soon be a thing of the past thanks to advancements in intra-oral scanners and cameras. In addition to being quicker and more accurate, these digital images also do away with all laboratory procedures, which greatly reduces the possibility of mistakes. Using AI, the computer can guide the dentist as they create a digital impression, optimize the final scan by eliminating the unwanted frames during scanning, automatically detect the margins of the preparations and develop a restoration design that imitate the anatomy of the lost tooth structure. ^98^ Computer Aided Design (CAD) Computer Aided Manufacturing (CAM) techniques, like subtractive milling, and additive manufacturing techniques, like 3D printing, are currently used to create prostheses. It has replaced the labor-intensive and challenging traditional casting process while dramatically lowering human error in the finished prosthesis. These technologies can also be employed to create precise orthodontic appliances. ^98^

AI software has aided in pre-surgical planning procedures to the smallest detail in oral surgery and implantology. The introduction of robotic surgery has made one of the most significant uses of AI in oral and maxillofacial surgery. Simulating human body motion and intellect is critical in robotics science. ^99^ However, the discipline of surgery has been revolutionized by artificial intelligence. The number of robotic surgeons doing semi-automated surgical procedures under the supervision of a skilled surgeon has increased significantly in recent years. Last but not least, one of the most cutting-edge uses of AI is found in the field of "bioprinting," which allows living tissue and even organs to be built into successively thin layers of cells. This technology could one day reconstruct oral hard and soft tissues lost due to pathological or unintentional causes. ^100^

### Dental Public Health

Artificial intelligence (AI) can be used in Dental Public Health, which involves diagnosing, preventing, and controlling dental diseases through research, education, and group dental care programs.

As an example, an AI-assisted diagnosis method is developed by employing Faster-Recurrent Convolutional Neural Network (RCNN) to predict the number and locations of caries lesions based on periapical films, which in the end, will help doctors diagnose the diseases with higher efficiency and precision. ^101^

### Endodontics

Although root canal configuration in a group of mandibular molars may seem similar, numerous unusual deviations may exist. ^73^ Cone-beam computed tomography (CBCT) has established the gold standard for reducing treatment failures due to these morphological variations and hence, optimizing endodontic therapy clinical results. ^102^ However, due to its increased radiation dosage compared to traditional radiography, CBCT is not widely employed. ^103^ To address such issues, AI has been applied to categorize the supplied data using a CNN to determine the anatomical variation in root canals. ^104^^,^^105^

### Oral and Maxillofacial Surgery

AI can be used in the diagnosis, surgical, and adjunctive treatment of disorders, diseases, injuries, and defects involving the functional and aesthetic aspects of the hard and soft tissues of the oral and maxillofacial regions and related structures.

AI technology has been widely applied for predicting facial attractiveness after orthognathic surgery. The above artificial intelligence models are built on either ANNs or CNNs. ^106^

Deep-learning-based algorithms can predict the virtual soft tissue profile after mandibular advancement surgery and compare its accuracy with the mass tensor model (MTM). ^107^

### Orthodontics and Dentofacial Orthopedics

ANNs have enormous potential to help in clinical decision-making. To get predictable outcomes for patients, orthodontic treatments must be meticulously planned. Teeth extractions, on the other hand, are not uncommon in orthodontic treatment plans. As a result, ensuring that the best clinical choice is taken before embarking on irreversible operations is critical. ^108^^–^^110^ Using DL for automated tooth segmentation on a 3D model of the jaw, the segmented teeth can be reconstructed with their roots from the CBCT using iterative closest point (ICP) algorithm to form a complete digital dental model, and to obtain the necessary information for orthodontic treatment simulation. ^111^^,^^112^

### Periodontics

There are two clinical categories of periodontitis, according to the American Academy of Periodontology's classification of periodontal disease in 1999: aggressive (AgP) and chronic (CP). Due to the disease's complex pathophysiology, no clinical, microbiological, histological, or genetic test can distinguish AgP patients from CP patients. ^113^ Many non-surgical and surgical techniques have been developed to manage periodontally compromised teeth and supporting structures. Despite advancements in treatment methods, there has been no major improvement in diagnosing and forecasting the prognosis of periodontally compromised teeth (PCT). ^114^ Clinical diagnosis and prognosis are highly reliant on empirical data. Deep CNN algorithms have shown a high potential for usefulness and accuracy in diagnosing and predicting the need for extraction of PCT. ^115^

### Oral and Maxillofacial Radiology

In detecting and identifying anatomical structures, CNNs have demonstrated promise. For instance, some people have been taught to identify and name teeth using periapical radiographs. When it comes to identifying and classifying teeth, CNN's accuracy rating of 95.8-99.45 percent closely resembles that of clinical specialists, whose accuracy score is 99.98 percent. Dental cavities can be found and diagnosed with CNNs as well. In 3000 periapical radiographs of posterior teeth, a deep CNN algorithm identified carious lesions with an accuracy of 75.5-93.3 percent and a sensitivity of 74.5-97.1 percent. ^116^ With sensitivity ranging from 19 percent to 94 percent; this represents a considerable advancement over the use of radiographs alone for diagnosis by medical personnel. When combined with their speed, deep CNNs have a high potential for improving the sensitivity of dental caries diagnosis, making them one of the most effective technologies in this field. ^117^^,^^118^ Another application of the AI in dental radiology is the use of artefact reduction algorithms that help the enhancement of the radiographic images and eliminate the distortion effect radio-opaque objects on the fine details of the acquired image, while reducing the need for high radiation dose and large voxel size. ^119^^,^^120^

### Oral Medicine and Pathology

Early identification and diagnosis of oral lesions are critical in dental offices since early detection improves prognosis greatly. Because certain oral lesions might be precancerous or cancerous, obtaining an accurate diagnosis and treating the patient appropriately. ^121^ CNN has been demonstrated to be a potential tool in diagnosing head and neck cancer lesions. CNN offers excellent promise for recognizing tumoral tissues in tissue samples or on radiographs, with specificity and accuracy of 78–81.8 percent and 80–83.3 percent, respectively (compared to specialists' 83.2 percent and 82.9 percent, respectively). ^117^

### Pediatric Dentistry

AI in Pediatric Dentistry can be used in the same way it is used in adults by providing primary and complete preventative and therapeutic oral health diagnosis, care, and consultation knowledge for infants and kids through adulthood.

AI has the potential to solve discrepancies that may arise throughout the analysis of growth data, and AR augmented reality methodologies have been developed to educate patients and families about growth disorders and their treatments. ^122^

### Prosthodontics

AI models serve as a dependable diagnostic tool for tooth shade selection, automated restoration design, mapping the preparation finishing line, optimizing manufacturing casting, predicting facial changes in patients with removable prostheses, and designing removable partial dentures. ^105^^,^^114^ (See Table 1) which summarizes a set of applications for dental activities that integrates AI technology.

**Table 1.** Summary of relevant studies on artificial intelligence applications in dentistry.

The Utilization of Artificial Intelligence in Dentistry			
Field of Research	AI Technique	Applications	Reference
Image segmentation	Convolutional neural networks (CNN)	Panoramic radiographs to test a novel method for automatic teeth segmentation	^143^^,^^144^
Radiology	CNN	Identification and classification of dental implant systems	^145^^,^^146^
Prosthodontics	Intrinsic AI	Tracing the margin line of the implant abutment	^147^^,^^148^
CT scans	CNN	To develop an automated mandible segmentation technique.	^149^^,^^150^
Panoramic radiographs	CNN	To evaluate the performance of a CNN for detecting osteoporosis	^151^^,^^152^
Orthodontics	Artificial neural networks (ANN)	Diagnosis of the need for orthodontic extraction	^108^^,^^153^
Periodontics	CNN	Diagnosis and prediction of periodontally compromised teeth	^154^^,^^155^
Oral medicine	ANN	To predict the occurrences of Bisphosphonate Related Osteonecrosis of the Jaw (BRONJ) associated with a dental extraction.	^156^^,^^157^
Dental periapical radiographs	CNN	To recognize and classify teeth position	^158^^,^^159^
Image segmentation	CNN and RNN	To develop a fully automated image analysis for mandible and anatomical landmark segmentation.	^160^^,^^161^
Dental Public Health	Fast R-CNN	Automatic teeth recognition, Craniomaxillofacial Landmark Detection	^162^^,^^163^
Endodontics	CNN	Score periapical lesion on an intraoral periapical radiograph For diagnosis and planning of treatment in endodontics	^164^^,^^73^
Oral and Maxillofacial Surgery	ANN, CNN, DL, MTM	Detection of ameloblastomas and keratocystic odontogenic tumors Diagnosing, treatment planning, and predicting the prognosis of orthognathic surgery	^165^^,^^166^
Oral Medicine and Pathology	ANN and CNN	Early oral cancer diagnosis, Dental image diagnosis	^167^^,^^168^^,^^144^
Oral and Maxillofacial Radiology	Deep CNNs	Dental and maxillofacial image analysis Detection and diagnosis of dental caries using a deep learning-based	^169^^,^^170^
Orthodontics and Dentofacial Orthopedics	CNN	Fully automatic segmentation of sinonasal cavity and pharyngeal airway based Accuracy detection of a posteroanterior cephalometric landmark	^171^^,^^172^
Pediatric Dentistry	Augmented Reality (AR)	To motivate oral hygiene practice in children: protocol for the development. Evaluation of holohuman application as a novel educational tool in dentistry	^173^^,^^174^
Periodontics	Deep CNN	Identify and classify dental implant systems using panoramic and periapical radiographs. Diagnosis and prediction of compromised teeth	^175^^,^^154^
Prosthodontics	ML	Oral and craniofacial imaging Tooth-supported fixed and removable prosthodontics	^176^^,^^114^
**Application of Artificial Intelligence in 3D Digital Dentistry**			
CAD/CAM	CNN	Estimate the debonding probability of CAD/CAM crowns	^177^
	Intrinsic AI and algorithms of CAD software	Automatically trace the margin line of the implant abutment through subgingival.	^178^
	ML models (RF, ET, LightBM, CBDT, and XGBoost)	Predict the flexural strength of CAD/CAM resin composite blocks (RCBs)	^179^
	CNN	CAD/CAM implant dentistry planning using three-dimensional cone-beam computed tomography (CBCT) images	^180^
3D printing	(ANN) supported by genetic algorithms (GA)	Optimization of the 3D-printing process in terms of features and material selection	^181^
	DL	Fabrication and maturation of 3D bioprinter tissues and organs	^182^
	DL-based PointNet++ model	3D Printing of Tooth Model	^183^
3D scanning	CNN	Enhancing the resolution of CT image assessment	^184^
	ML	Diagnosis and planning in plastic and reconstructive surgery	^185^
	CNN	Tooth segmentation	^186^
	Generative adversarial network	Tooth segmentation	^187^
	CNN	Automated tooth labeling	^188^

### AI Applications in 3D Digital Dentistry

Digital dentistry has changed the dental industry by introducing fresh methods, tools, and interactions. Research directions and opportunities for material scientists have been prompted by innovation. Many digital processes for production processing, particularly computer-aided designing and manufacturing (CAD/CAM), have already been integrated into dental treatment procedures. ^123^^–^^126^

**Figure 7.**
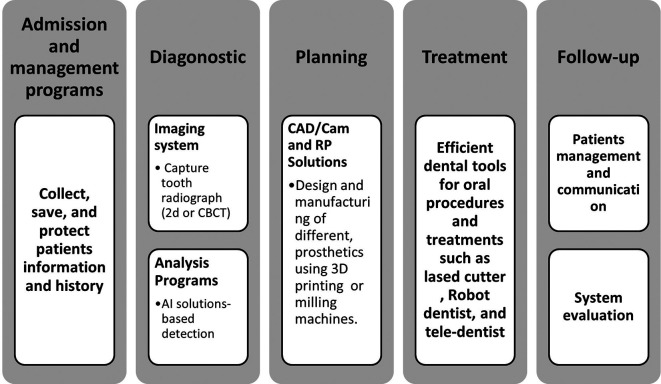
The process of digitalizing the dental system.

Nowadays, the advances in Artificial Intelligence (AI) and 3D imaging systems make dentists rely more and more on digital technologies for detection and treatment. ^127^^,^^128^ Adopting AI-based digital technology for clinical solutions and dental laboratories allows the dentist to digitally repeat the whole procedure and treatments. Furthermore, assure sharing of information across the entire process of the digital dental system, shown in Figure 7. Using a digitalized dental system brings real advantages in accuracy and quality, is time-consuming, and decreases costs. ^129^

Digital dentistry now incorporates diagnostics, decision-making, treatment conduct, and re-evaluation of lifelong management of patient's oral health, as well as computer-aided designing and manufacturing (CAD/CAM) and rapid prototyping fields.

Rapid prototyping (RP) is a subset of rapid manufacturing and a type of (CAM). It is a method of creating three-dimensional (3D) models of a finished product or a component of a larger whole using 3D printers quickly and automatically. The recent revolutionary application of 3D scanning and printing is AI-based orthodontic treatment. ^130^^–^^132^ AI is applied in orthodontics at all stages, from diagnosis to treatment planning and follow-up supervision. Where the Aligners and treatments can be designed with the help of these 3D scans. After the aligners are printed, a data algorithm is developed that intelligently specifies how the teeth should be managed to move, the amount of pressure that should be applied, and sometimes even recognizes the pressure points for that particular tooth. ^133^

Large dental labs in Europe and USA have increasingly implemented CAD/CAM technology. Recent advancements in 3D printing have broadened its applications that involve dental models, orthodontic appliances, mouth guards, and appliances for guided implant surgery.

All CAD/CAM systems are made up of three parts:

• 3D Scanners convert geometric features into digital data that a computer can process.
• CAD is a data processing software that produces a dataset for the component to be manufactured, depending on the application.
• CAM is a manufacturing technology that transforms a dataset into the desired component.

CAD/CAM technology has revolutionized the concept of dentistry. Standardized production processes have significantly improved the quality of dental prostheses. This provides extremely effective quality management. It enhanced productivity significantly and changed dental laboratories from manufacturers into sophisticated computerized production centers. Increased productivity. In addition, it leads to a competitive capability to make dental prostheses independent of the manufacturing site, which could be a crucial reason for high-wage countries to maintain business volume in the country. ^134^ Lastly, CAD/CAM technology has enabled the high-precision machining of novel materials such as high-performance ceramics and titanium.

Recently, researchers have been looking into ways to improve the accuracy of 3D scanning, CAD\CAM, and 3D printing, such as using AI models. This can save money and time and reduce the number of visits required for precise fitting. Table 1 includes a summary of the applications of AI in 3D digital dentistry during recent years.

Even so, some drawbacks of this manufacturing technology should be handled. The rising cost of machines may exceed the limits of smaller laboratories. Additionally, some applications are limited due to software and manufacturing procedures. CAD/CAM technology has already changed dentistry and will gradually replace traditional methods of fabricating dental restorations.

## Strengths and Weaknesses of AI in Dentistry

### Strengths of AI in Dentistry

AI enables the integration of many heterogeneous data domains, such as patient data, clinical knowledge of the patient, imaging results, and so on, creating the greatest use of these data obtained and understanding their contact. Artificial intelligence-based supports discoveries by supplementing other research levels and current modeling methodologies within silico experimentation choices in traditional research hierarchies.

As previously noted, AI may simplify mundane tasks and enhance the contact time between patient and doctor. This might include not just diagnostic aid technology but also data from patients' voices and speech to help the dentist keep track of the record and help in saving time. ^135^

Using these continually obtained data will reduce the effect of "on-off-medicine," in which patients only meet the doctor for a few seconds. However, the health issues develop over the year leading to an increase and decrease in the symptoms of the disease over time. Continuous and constant health and behavior analysis will allow for a far deeper, personalized knowledge of the underlying drivers and processes of health and illness. ^136^

### Weaknesses of AI in Dentistry

Diagnostic and treatment expenses are expected to reduce due to betterment in the healthcare field by embedding AI in the system resulting in more early diagnoses of chronic diseases. However, the disadvantage of the use of AI in dentistry includes. ^137^

AI may also help alleviate labor scarcities, which have been noted and are projected to remain in many regions of the world, therefore assisting in achieving the sustainable development goals defined by WHO.

AI-based apps will expedite treatment, freeing the dental staff of time-consuming, mundane duties, improving health at a cheaper cost for a larger population, and eventually enabling customized, predictive, preventative, and participative dentistry. However, AI solutions have not yet been widely adopted in routine dental practice, owing to fewer data and development standards and ethical concerns. ^34^

**Figure 8.**
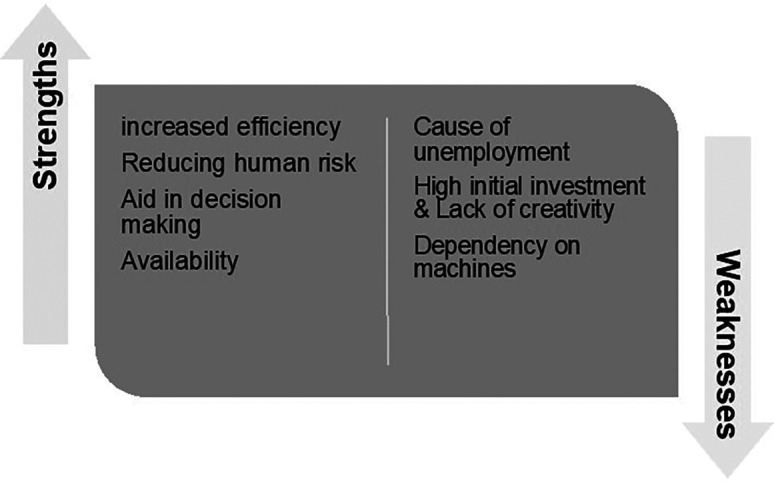
Strengths and weaknesses of AI.

The misuse of data and security concerns of AI is other important parameter in dentistry. relying entirely on the machine to decide the health care services is critical, and entrusting a machine will not be okay regarding human health. It would be suitable only if AI provides low cost with excellent patient benefits and, as a result, improve society. ^138^ Figure 8 provides of summary of the strengths and weaknesses of AI within the dentistry area.

## The Main Challenges of using AI in Dentistry

Clinical data storage and interchange present significant challenges when using AI algorithms in the healthcare industry. The initial training of the AI system and further training, validation, and improvement require patient-specific data. Additionally, the development of AI will promote data flow within different businesses and, in some circumstances, across international boundaries. When integrating AI into healthcare operations, systems must be changed to protect patient privacy and confidentiality. Personal data must therefore be anonymized before further distribution is considered. Despite being able to implement these precautions, the healthcare industry is still dubious about secure data sharing. ^139^

AI systems raise safety issues as well. It is necessary to create controls for the effectiveness of AI algorithms. The US Food and Medicine Administration created a new drug classification called "Software as Medical Device" to address this problem, ^140^ which is in charge of ensuring patient safety and safe innovation. Ambiguous responsibility in applying AI technologies is a further cause for concern. The replacement of people with autonomous agents raises a number of legal and ethical concerns because the legal system is based on the core tenet that blame and crime are ultimately attributed to people. For the foreseeable future, these challenges will continue to pose a significant challenge to the judicial system. ^141^

The openness of AI algorithms and data is a major concern. The precision of the annotations and labeling of the training dataset significantly influences the quality of predictions generated by AI systems. Poor outcomes may stem from wrongly tagged data. Clinic-labeled datasets could be of different quality, making the developed AI systems less effective. Additionally, healthcare professionals should be able to argue the results and projections of an AI system and have a complete understanding of them. The interpretability of AI technology is a well-known problem, and much advancement is required before some algorithms, like neural networks, can provide clinical diagnoses or treatment recommendations with total transparency. ^142^

## Conclusion

In the recent decade, the field of AI has evolved enormously. While breakthroughs in AI, such as neural networking, natural language processing, image identification, and speech recognition, have revolutionized medicine and dentistry in many ways, they are not without downsides and obstacles. One example is the high upfront capital equipment expenses. When applied to clinical medicine, the methods sought for adoption in the medical profession have certain severe limitations. At some point, simple flowcharts and statistical pattern matching become unmanageable. Algorithms frequently make incorrect assumptions, leading to doubt regarding their responsibility in healthcare. Although AI systems are a big help in the field of dentistry and dental education, biological processes are far more complicated, and AI systems will never be able to replace human knowledge, competence, and decision-making capacity.

Despite numerous studies showing potential applications of AI in dentistry, these systems are far from being able to completely replace dental professionals. AI should be viewed as an additional benefit for dentists and other professionals. To ensure that humans can continue to oversee the care and make informed judgments in dentistry, AI must be implemented in a safe and controlled manner. Most institutions are currently unprepared for the duty of providing dental and continuing education training, which is necessary for effective AI integration in dentistry. Additionally, augmented reality (AR) and virtual reality (VR) both benefit from AI. Mixed reality is a novel concept that incorporates parts of generative AI, VR, and AR into computer-superimposed information overlays to improve learning and surgical planning. As many AI systems for various dentistry disciplines are being researched and have yielded promising early results, a future for AI in the healthcare system cannot be ruled out. AI systems offer significant promise as a valuable tool for oral health practitioners.

The results of this review indicate that embracing AI-based technologies for clinical solutions and dental laboratories enables dentists to digitally repeat procedures and treatments and ensure data sharing throughout the entire digital dental system process. Furthermore, it provides significant benefits in accuracy and quality, as well as saving time and money.
