# Supplementary material for: Contemporary Role and Applications of Artificial Intelligence in Dentistry
Source: F1000Res. 2023 Sep 20;12:1179. [Version 1] doi: 10.12688/f1000research.140204.1 (PMC10630586; doi:10.12688/f1000research.140204.1)
Supplement: Peer review history

# Reviewer report: Reviewer response for version 1

**DOI:** 10.5256/f1000research.153536.r212090

**Published:** 2023-11-07

**Authors:** Dipmala Salunke

**Affiliations:** 1 JSPM’s Rajarshi Shahu College of Engineering, Pune, Maharashtra, India

**Summary of the article:**

 The article provides a detailed exploration of the role of Artificial Intelligence (AI) in dentistry, its potential applications, and its limitations. It covers a range of topics, including AI algorithms, the pros and cons of using AI in dental specialties, and the need for AI to complement rather than replace dental professionals. The article also discusses the integration of AI with augmented and virtual reality technologies in dentistry.

**Strengths of the Article:** 

 Comprehensive Coverage: The article comprehensively explores the potential applications of AI in various dental specialties and discusses both the advantages and limitations of AI in the field.

 Clarity of Purpose: The article clearly states its purpose, which is to assess the advancement of AI algorithms in dentistry and their performance in diagnosis, clinical decision-making, treatment, and prognosis prediction.

 Integration with Emerging Technologies: The discussion of AI's integration with augmented reality and virtual reality is valuable, as it highlights innovative approaches to dental education and surgical planning.

 Real-World Applications: The mention of AI's potential benefits in clinical solutions and dental laboratories demonstrates the practical advantages of AI in the dental field, such as data sharing, accuracy, and cost savings.   

**Areas for Improvement:**

****

 Ethical Considerations: While the article touches on the limitations of AI, it could expand on ethical considerations in more detail, such as patient data privacy, potential biases in AI algorithms, and the need for human oversight in clinical decision-making.

 Illustrations: Authors are suggested to include images of good resolution specially, figure number 1.

**Conclusion:**

 The article “Contemporary Role and Applications of Artificial Intelligence in Dentistry”, offers a promising exploration of AI's impact on dentistry.However, it could be enhanced by addressing topics such as patient data privacy, potential biases in AI algorithms, and the essential role of human oversight in clinical decision-making.The content of the article is not only informative but also relevant, especially for readers who are starting research in the field of dentistry, therefore, I wholeheartedly recommend accepting this manuscript for publication with reservations outlined above.

Is the review written in accessible language?

Yes

Are all factual statements correct and adequately supported by citations?

Yes

Are the conclusions drawn appropriate in the context of the current research literature?

Yes

Is the topic of the review discussed comprehensively in the context of the current literature?

Yes

Reviewer Expertise:

Dental image processing, Deep learning, AI

I confirm that I have read this submission and believe that I have an appropriate level of expertise to confirm that it is of an acceptable scientific standard, however I have significant reservations, as outlined above.

# Reviewer report: Reviewer response for version 1

**DOI:** 10.5256/f1000research.153536.r212091

**Published:** 2023-10-26

**Authors:** Hemant Sawhney

**Affiliations:** 1 Sharda University, Noida, Uttar Pradesh, India

**Summary:**

 The manuscript titled "Contemporary Role and Applications of Artificial Intelligence in Dentistry" provides a comprehensive overview of the current state of artificial intelligence (AI) in the field of dentistry. The author discusses various applications of AI, from diagnosis and treatment planning to predictive analytics, virtual consultations, and the use of robotics in dental surgery. Overall, the paper is well-structured and informative, making a valuable contribution to the field of AI in dentistry.

**General Comments:**

1. **Clarity and Organization:** The manuscript is well-organized, with a clear introduction and structured sections that make it easy for readers to follow the discussion. 
2. **Citations and References:** The paper adequately cites relevant sources to support the discussed points. 
3. **Depth of Coverage:** The paper covers a wide range of topics related to AI in dentistry, providing a comprehensive overview. However, it might be beneficial to delve deeper into specific case studies or examples of successful AI applications in dentistry to offer a more practical understanding for readers.
4. **Technical Accuracy:** The manuscript appears to be technically accurate, and the concepts related to AI are well-explained. It would be helpful if the author could provide a glossary or definitions for any technical terms that might be unfamiliar to the general reader.

**Specific Comments:**

1. **Case Studies and Examples:** The manuscript could benefit from the inclusion of specific case studies or real-world examples of AI applications in dentistry. This would illustrate the practical benefits of AI to the readers.
2. **Ethical and Legal Considerations:** It would be valuable to address the ethical and legal considerations surrounding AI in dentistry, such as patient data privacy, liability, and informed consent. This would provide a more well-rounded perspective on the topic.
3. **Future Trends:** Discussing future trends and potential advancements in AI within the field of dentistry could be an excellent addition. This would give the paper a forward-looking perspective and help readers understand the evolving landscape.

**Conclusion:**

 The manuscript, "Contemporary Role and Applications of Artificial Intelligence in Dentistry," provides a valuable and informative overview of AI applications in dentistry. To enhance the paper, I recommend including case studies, addressing ethical considerations and discussing future trends.  With these improvements, the manuscript will offer a more comprehensive understanding of the topic.

 Overall, this paper contributes to the growing body of knowledge on AI in dentistry and should be considered for publication with the suggested revisions.

**Recommendation:** I recommend accepting this manuscript with minor revisions.

Is the review written in accessible language?

Yes

Are all factual statements correct and adequately supported by citations?

Yes

Are the conclusions drawn appropriate in the context of the current research literature?

Yes

Is the topic of the review discussed comprehensively in the context of the current literature?

Yes

Reviewer Expertise:

Artificial Intelligence in Dentistry and Radiology, Oral Medicine, Maxillofacial Radiology

I confirm that I have read this submission and believe that I have an appropriate level of expertise to confirm that it is of an acceptable scientific standard.

# Reviewer report: Reviewer response for version 1

**DOI:** 10.5256/f1000research.153536.r209464

**Published:** 2023-10-17

**Authors:** Reham Osman

**Affiliations:** 1 Department of Prosthodontics, Cairo University, Giza, Giza Governorate, Egypt

The article at hand had described the concept of AI and its different types, its application in health care sector in details and some of its applications in dental field. As well, the article has highlighted the strength and weakness point of technology and came up with some future recommendations. 

 The reviewer thinks that a huge effort has been made in writing this review and that it is recommended for publication after addressing all of the below mentioned points.

**The title “Application of AI in Dentistry** **”**

- Different paragraphs beneath this title are more towards describing and explaining the application of AI in general health sector generally rather than in dentistry as the title entails;
- example “With the advent of huge scientific data, the diagnosis of human congenital abnormalities has improved. In medical research, the support vector machine (SVM), a machine-learning model, has become a typical way of analysis. SVM may be used to classify complex situations like traumatic brain damage. ^60, 61^DL may play a crucial role in advancing cardiovascular medicine due to the requirement for precision in diagnosis and therapy. ^62^ With the adoption of deep learning, it will be feasible to identify numerous cardiovascular disorders with high-precision diagnosis. ^63^ Wearable technology combined with intelligence applications can anticipate a patient's life-threatening crisis such as a stroke, allowing physicians to deliver effective, early therapies. ^54^ Using AI to examine viruses has boosted transitional studies in viral immune monitoring.”  This paragraph should be shifted to the title above it “Artificial intelligence for health care”.

**The title:** **State-of-the-art AI technology in Dentistry**

- Same comment as in previous point. The whole paragraph under this title seems to be describing more the components and different types of artificial intelligence rather than describing Art of AI in dentistry.  
- Reviewer would suggest that the authors rename this paragraph differently so it is not misleading to reader.

**“Taking a dental impression might soon be obsolete due to advancements in intra-oral scanners and cameras. These digital impressions minimize all laboratory operations, which sharply lowers the frequency of errors and is quicker and more accurate.** **”**

- This is a very sharp statement not supported by current state of art in literature. Digital impressions are effective and comparable to conventional impressions in cases of unilateral partially dentate patients with up to 4 missing units. However, in fully edentulous cases and extensive cases crossing midlines, accuracy of digital impressions is inferior to their conventional counterparts. Digital impression acquisition is challenging in fully edentulous cases because of lack of anatomic landmarks that allow images stitching during the scan process besides mobility of soft tissue especially floor of mouth of arch during scan process of mandibular arches.  All of these factors cast a shadow about the possibility of digital impression obliterating the need for conventional impression.

**“** **Taking a dental impression may soon be a thing of the past thanks to advancements in intra-oral scanners and cameras. In addition to being quicker and more accurate,** **”**

 Same comment as mentioned in point above

 The reviewer suggests that the authors either modify and rephrase this statement as per current state of literature and add necessary references and citations or totally delete it.

- In image six, there is a typo error in post-appointment bullets: should be “AI Clinician evaluation”.
- Resolution of image 3 & image 5 is low, and images are not clear and cannot be easily read, please re-provide with a better resolution one that could be easier read.
- Under the title: “Weakness of AI in Dentistry”.
- The first statement in the paragraph “Diagnostic and treatment expenses are expected to reduce due to betterment in the healthcare field by embedding AI in the system resulting in more early diagnoses of chronic diseases.”  is considered an advantage rather than a disadvantage or a weakness.
- The last statement in the first paragraph “However, the disadvantage of the use of AI in dentistry includes” The sentence is incomplete and it is not mentioned what is the disadvantage of using AI in Dentistry.
- Under the title: “oral medicine and pathology”

The statement “Because certain oral lesions might be precancerous or cancerous, obtaining an accurate diagnosis and treating the patient appropriately.” The statement is not complete and need to be adjusted.

- Can the authors please add a reference for this statement: “AI may also help alleviate labor scarcities, which have been noted and are projected to remain in many regions of the world, therefore assisting in achieving the sustainable development goals defined by WHO.”
- This Statement “AI may also help alleviate labor scarcities, which have been noted and are projected to remain in many regions of the world” under the weakness of artificial intelligence title is the opposite of increasing the unemployment which is mentioned in Figure 8 and which is properly described as one of AI weakness in health care sector.
- Under the title: “AI application in 3D digital dentistry”

“Using a digitalized dental system brings real advantages in accuracy and quality, ***is time-consuming***, and decreases costs.” Can the authors please check this sentence that one of the advantages of digital dentistry is time consumption?

- “This provides extremely effective quality management. It enhanced productivity significantly and changed dental laboratories from manufacturers into sophisticated computerized production centers. ***Increased productivity***.” Please delete increased productivity as it does not form a comprehensible sentence.
- “Lastly, CAD/CAM technology has enabled the high-precision machining of novel materials such as high-performance ceramics and titanium.” Can the authors please add a reference for this statement.

FINAL GENERAL RECOMMENDATION: that the authors alter the title of paper into “Contemporary Role and Applications of Artificial Intelligence in Health care sector with specific interest in dental applications”. It is in the reviewer’s opinion will better fit the content of the paper more than the current title. Further, the applications of AI in different fields of dentistry are very briefly described considering

Is the review written in accessible language?

Yes

Are all factual statements correct and adequately supported by citations?

Partly

Are the conclusions drawn appropriate in the context of the current research literature?

Partly

Is the topic of the review discussed comprehensively in the context of the current literature?

Yes

Reviewer Expertise:

Prosthodontics, Oral implanvology, Digital Dentistry, 3D- printing, Biomechanics

I confirm that I have read this submission and believe that I have an appropriate level of expertise to confirm that it is of an acceptable scientific standard, however I have significant reservations, as outlined above.
